# Transcarotid vascular access for transcatheter aortic valve implantation: is choosing the left side always right?

**DOI:** 10.1186/s13019-024-02661-7

**Published:** 2024-04-10

**Authors:** Adil Salihu, David C. Rotzinger, Guillaume Fahrni, Anna Nowacka, Panagiotis Antiochos, Stephane Fournier, Olivier Muller, Matthias Kirsch, Henri Lu

**Affiliations:** 1 Division of Cardiology, Lausanne University Hospital and University Hospital, Rue du Bugnon 46, Lausanne, 1011 Switzerland; 2 Department of Diagnostic and Interventional Radiology, Lausanne University Hospital and University Hospital, Lausanne, 1011 Switzerland; 3 Division of Cardiovascular Surgery, Lausanne University Hospital and University Hospital, Lausanne, 1011 Switzerland

**Keywords:** Computational vascular modeling, TAVI, TAVR, Transcarotid, Vascular access, Vessel tortuosity

## Abstract

**Background:**

The transcarotid (TC) vascular access for transcatheter aortic valve implantation (TAVI) has emerged as the first-choice alternative to the transfemoral access, in patients unsuitable for the latter. The use of both the left and right common carotid arteries (CCAs) for TC-TAVI has been described, but the optimal side is subject to debate. We conducted this pilot study to compare the level of vessel tortuosity and plaque burden from either the left CCA to the aortic annulus, or the right CCA to the aortic annulus, considering them as surrogates for technical and procedural complexity.

**Methods:**

Consecutive patients who underwent TC-TAVI between 2018 and 2021 in our institution were included. Using three-dimensional reconstruction, pre-TAVI neck and chest computed tomography angiography exams were reviewed to assess the tortuosity index (TI), sum of angles metric, as well as plaque burden, between each CCA and the aortic annulus.

**Results:**

We included 46 patients who underwent TC-TAVI. No significant difference regarding the mean TIs between the left and right sides (respectively 1.20 and 1.19, *p* = 0.82), the mean sum of angles (left side: 396°, right side: 384°, *p* = 0.27), and arterial plaque burden (arterial plaque found in 30% of left CCAs and 45% of right CCAs, *p* = 0.19) was found.

**Conclusions:**

We found no convincing data favoring the use of one particular access side over the other one. The choice of the CCA side in TC-TAVI should to be made on a case-by-case basis, in a multidisciplinary fashion, and may also depend on the operators’ experience.

## Introduction

During the past twenty years, transcatheter aortic valve implantation (TAVI) has emerged as the primary procedure for treating symptomatic severe aortic stenosis in patients aged ≥ 75 or those who are younger but at high surgical risk [1]. As a result, the number of TAVI interventions has considerably increased, and this trend is expected to continue in the next decade [2].

While the transfemoral (TF) vascular access is considered as the gold-standard pathway for TAVI, it is not suitable for up to 10% of patients, mainly due to anatomical contraindications, such as small or heavily calcified iliofemoral vessels or extreme vessel tortuosity [3].

Several alternative vascular accesses have been developed for these specific settings, including the transcarotid (TC) approach [4]. In this procedure, the common carotid artery (CCA) is surgically exposed and punctured, allowing for the insertion of the transcatheter heart valve. The latter is then descended through the brachiocephalic trunk and part of the aortic arch to the level of the aortic annulus, where it is released [5]. The TC approach is interesting as it avoids the need for thoracotomy and provides a direct and short pathway to the aortic valve, with the benefit of stable catheter delivery and improved movement precision [6]. Several studies have suggested that it might yield outcomes comparable to the gold-standard TF access [7, 8], and thus, could be considered as the first-line alternative when TF-TAVI is unsuitable [3]. Despite the overall good results with TC-TAVI, some aspects of the procedure remain unknown or subject to debate, such as the preferred side (left or right) of the CCA to access [9]. In fact, the use of both CCAs has been described, with a preference for the left CCA by many teams. In a previous meta-analysis, we showed that approximately 70% of procedures were performed through this artery [3]. Reasons why one side would be preferred over the contralateral one are not clear, and local experience may play a role in this decision.

Using pre-intervention neck and thoracic computed tomography angiography (CTA) exams performed in patients who underwent TC-TAVI in our institution, we conducted this retrospective pilot study to compare the level of vessel tortuosity and plaque burden between the left CCA and the aortic annulus, as well as between the right CCA and the aortic annulus. We considered these factors as surrogates for technical and procedural complexity.

## Materials and methods

### Study population

All patients with severe symptomatic aortic stenosis who underwent TC-TAVI at Lausanne university hospital (*Centre hospitalier universitaire vaudois*) between January 1st 2018 and December 31st 2021, and belonged to the SWISS TAVI Registry were retrospectively included in our study. All candidates for TAVI had a neck and chest CTA before intervention. Patients’ baseline characteristics, peri-procedural and post-procedural data were prospectively collected in a dedicated database. For all cases, suitability for TAVI and the choice of vascular access were assessed by a Heart Team, consisting of at least an interventional cardiologist, an echocardiographer, a cardiac surgeon and an anesthesiologist. TF-TAVI was the preferred approach unless patients met specific exclusion criteria, such as iliofemoral atherosclerosis, small or heavily calcified vessels (< 6 mm), mural thrombus, extreme vessel tortuosity, or abdominal aortic aneurysms. In such cases, TC-TAVI was considered feasible unless there were contraindications like small vessel diameter (< 6 mm), prior ipsilateral carotid artery intervention, heavy artery calcification and tortuosity, or significant stenosis (> 50%) or occlusion of the contralateral carotid artery. Transapical and transaortic approaches were reserved as the last alternatives if TF and TC approaches were not feasible.

### Ethical statement

All patients belonged to the SWISS TAVI Registry and provided written informed consent for the use of their data for research purposes. Our study was conducted in accordance with the principles of the Declaration of Helsinki. Ethical approval was given by the Vaud Canton ethics committee (*Commission cantonale d’éthique de la recherche sur l’être humain*), decision CER-VD 211/13, dated May 10th, 2013.

### CTA image acquisition and analysis

We conducted CTA image acquisitions using 256-row multidetector CT systems (Revolution CT, GE Healthcare, Milwaukee, USA). Patients were positioned lying on their back and instructed to raise their arms above their head while holding their breath for image acquisition. Non-contrast ECG-gated images centered on the heart were first acquired to derive the aortic valve calcium score (these series were not analyzed as part of this study). In the subsequent step, ECG-gated CT angiography of the carotid arteries, aorta, and iliac arteries was performed in the craniocaudal direction. This was accomplished by administering 100 mL of iodinated contrast medium (350 mg/mL, Accupaque 350, GE Healthcare) through an antecubital vein (preferably on the right side). Two radiologists independently evaluated the images, and any disagreement was resolved by consensus.

**Fig. 1:**
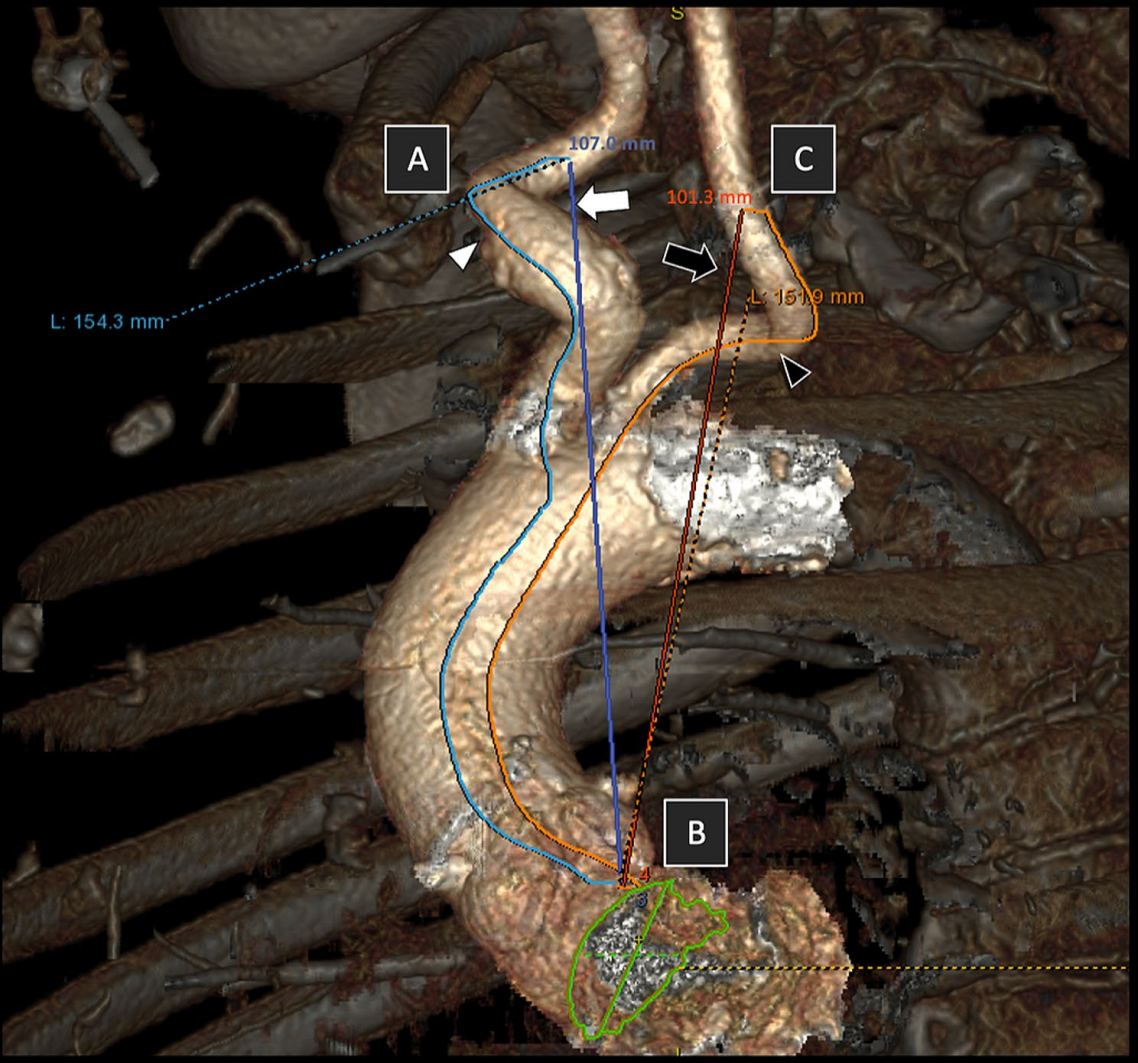
3D reconstruction of the aortic arch with the right brachiocephalic trunk and CCA, and left CCA. Point **A**: theorical surgical puncture site on the right CCA, located 2 cm distal to the brachiocephalic trunk bifurcation. Point **C**: same level as point A, located on the left CCA. Point **B**: Located on the aortic annulus (green circle). Centerlines (right CCA: light blue line, left CCA: orange line) and direct lines (dark blue and red lines) are drawn between, respectively, Point **A** and Point **C**, and Point **B** and Point **C**

Multiplanar, curvilinear and three-dimensional volume rendering reconstructions of the aortic arch and carotid arteries were generated using the AW-server software (version 3.2, GE Healthcare, Buc, France). Measurements were taken between a specific point on the CCAs (located 2 cm distal to the bifurcation of the brachiocephalic trunk for the right side, point A in Fig. 1, with a corresponding point at the same level for the left side, point C), and the center of the aortic annulus (referred to as point B). Point A represents the approximate site of surgical puncture on the CCA during TC-TAVI.

### Outcomes

#### Tortuosity index

To assess the level of vessel tortuosity, we employed two parameters. The first parameter was the tortuosity index (TI), which is calculated as the percentage ratio of actual length of the arterial segment (determined from curvilinear reconstructions) divided by the shortest distance between the two points (as shown in Fig. 1, point A to point B) [10]. The TI has been previously used to assess arterial tortuosity in various conditions, including aortic diseases [11, 12]. In our study, the shortest distance between points A and B was measured using calipers on three-dimensional volume rendering reconstructions. A TI value of “1” indicates no tortuosity and as the value increases, the vessel is considered more tortuous. Inter-observer reproducibility was not tested, based on the results of a previous study [13].

#### Sum of angles metric

**Fig. 2:**
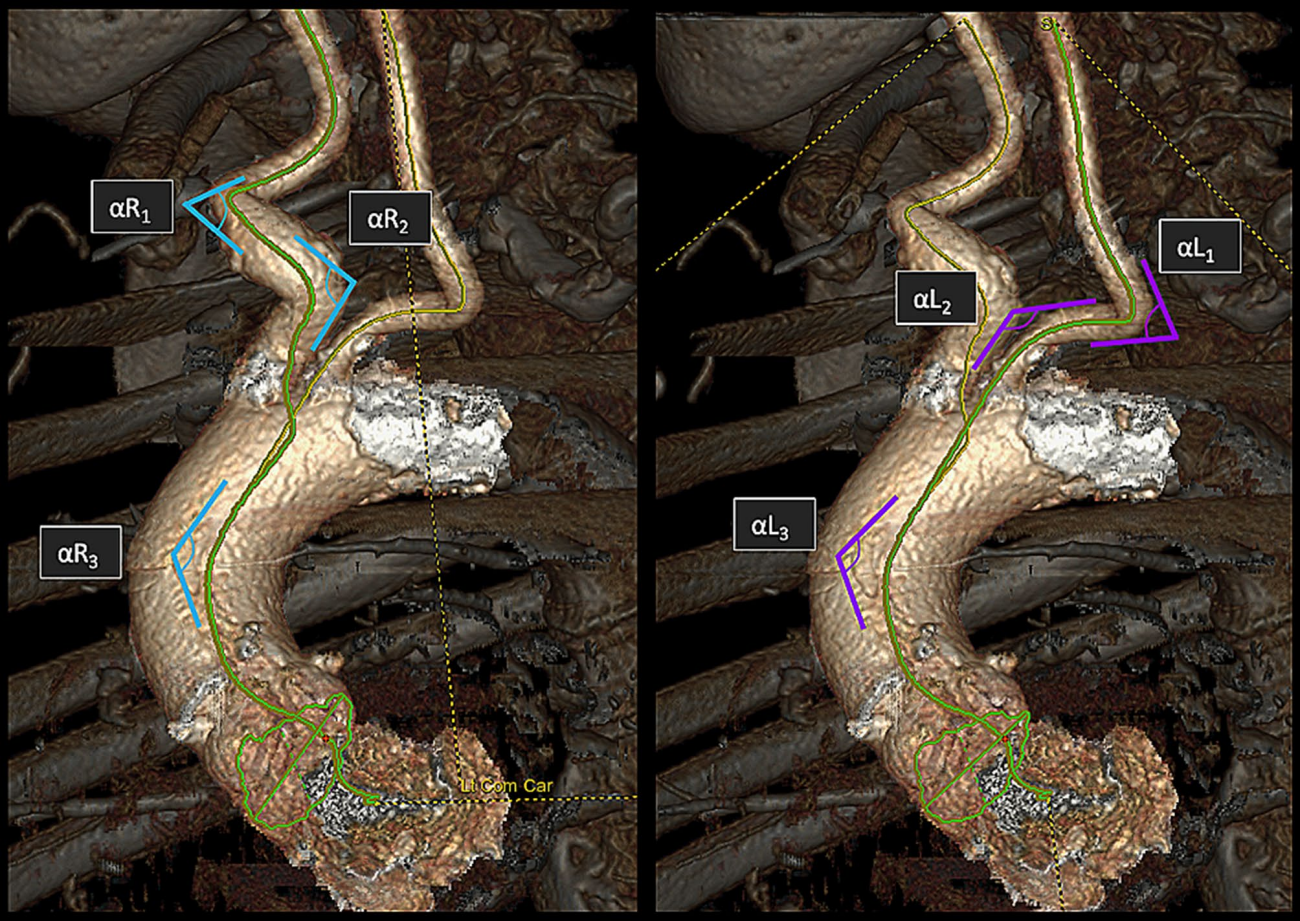
Measure of the sum of angles from the right and left CCAs to the center of the aortic annulus (respectively left and right panels). In both cases, three angles (respectively αR1, αR2, αR3 and αL1, αL2, αL3) were measured

The second method used to evaluate vessel tortuosity was the sum of angles metric. This approach involved measuring the angles of concave curvatures in a three-dimensional manner for each vessel. For example, in Fig. 2, the relevant angles between each CCA and the aortic annulus were first assessed. Each angle was then measured, and angulation was categorized as severe (< 30°), moderate (30–60°) or mild (> 60°), based on a previously established classification [12, 14]. The measured angles for each side were summed, with a smaller sum of angles indicating a more tortuous pathway. In cases where one side had two angles and the other one had three angles, 180° were added to the first side to ensure comparability. It is important to note that the sum of angles metric may be subject to inter-observer variability and should be interpreted with caution [11].

#### Plaque burden

Plaque burden was determined by the presence of any arterial plaque on the vessels, irrespective of the number or type (calcified, mixed, or non-calcified) of the plaques. The assessment did not involve quantification of the stenotic effect of the plaque (> 50% stenosis), unless it was considered clinically relevant.

#### Procedural characteristics and post-procedural clinical outcomes

Outcomes were reported according to the Valve Academic Research Corsortium (VARC-2) definitions [15]. The data collection period occurred before the publication of the more recent VARC-3 criteria [16], so those criteria were not used in our analysis.

### Statistical analysis

Categorical variables are presented as frequencies and percentages, and were analyzed using Pearson’s χ2 test. Continuous variables were tested for normal distribution using the Shapiro-Wilk test. For normally distributed variables, means with standard deviations (SDs) were reported, while for non-normally distributed variables, medians with interquartile range (IQR) were provided. Student’s t-test was used to compare normally distributed continuous variables, while the Mann-Whitney test was used for non-normally distributed continuous variables. A p-value of 0.05 was considered statistically significant. All statistical analyses were conducted using the SPSS 24.0 software (IBM Corp. Released 2016. IBM SPSS Statistics for Macintosh, Version 24.0. Armonk, NY: IBM Corp., USA).

## Results

### Patient characteristics

Between January 1st 2018 and December 31st 2021, a total of 46 patients underwent TC-TAVI, all of whom were included in our study. The baseline clinical and echocardiographic characteristics of the patients are summarized in Table 1. The mean age at the time of intervention was 80 ± 6 years, and 50% of the patients were women. The prevalence of key cardiovascular risk factors was as follows: diabetes mellitus (24%), hyperlipidemia (59%), hypertension (80%). Additionally, 17% of the patients had a history of lower extremity artery disease, while 41% of patients had a history of coronary artery disease. The mean left ventricle ejection fraction was 60 ± 12%, with an aortic valve area of 0.72 ± 0.16 cm^2^ and a mean gradient of 37 ± 11 mmHg.

**Table 1:** Patients baseline clinical and echocardiographic characteristics

Patients	*N* = 46
Age, years, mean +/- SD	80 +/- 6
Male gender	23 (50.0)
BMI kg/m^2^, mean +/- SD	26 +/- 4.8
**Cardiac comorbidities**	
Coronary artery disease	19 (41.3)
Previous PCI	6 (13.0)
Previous cardiac surgery	6 (13.0)
Pacemaker	3 (6.5)
**Other comorbidities**	
Diabetes mellitus	11 (23.9)
Hypertension	37 (80.4)
Hyperlipidemia	27 (58.7)
LEAD	8 (17.4)
Stroke	8 (17.4)
STS score, mean +/- SD	3.6 +/- 2.6
EuroSCORE II, mean +/- SD	3.9 +/- 3.1
**Echocardiographic characteristics**	
LVEF, percentage (%), mean +/- SD	60 +/- 12
Mean aortic gradient, mmHg, mean +/- SD	37 +/- 11
Aortic valve surface, cm^2^, mean +/- SD	0.72 +/- 0.16

Results are expressed as n (%), unless specified otherwise. SD: standard deviation, BMI: body mass index, PCI: percutaneous coronary intervention, LEAD: lower extremity arterial disease, STS: Society of Thoracic Surgeons, LVEF: left ventricular ejection fraction

### Outcomes

There was no statistically significant difference observed in the mean TIs between the left and right sides (respectively 1.20 and 1.19, *p* = 0.82). Similarly, there was no significant difference regarding the mean sum of angles of the left side (396°) and the right side (384°, *p* = 0.27), or the mean number of angles between the aortic annulus and the CCAs (left side: 2.47, right side: 2.59, *p* = 0.25). The majority of angles on both sides exhibited mild angulation (99.1% for the left side and 98.3% for the right side, *p* = 1.00). There was no significant difference between the left and right CCAs in terms of mean minimum diameters (respectively 6.57 mm and 6.74 mm, *p* = 0.92) and mean maximum diameters (respectively 7.29 mm and 7.55 mm, *p* = 0.72). An arterial plaque was detected in 30% of left CCAs and 45% of right CCAs, with no significant difference (*p* = 0.19). Only one patient had a plaque in the left CCA that caused a 50% arterial stenosis, while the other plaques did not result in a clinically-meaningful stenotic effect. Results are summarized in Table 2.

**Table 2:** Computed tomography angiography characteristics

	Left CCA (*n* = 46)	Right CCA (*n* = 46)	P value	
Minimum diameter, millimeter, mean +/- SD	6.57 +/- 1.00	6.74 +/- 0.97	0.92	
Maximum diameter, millimeter, mean +/- SD	7.29 +/- 0.96	7.55 +/- 1.14	0.79	
Tortuosity index, mean +/- SD	1.20 +/- 0.11	1.18 +/- 0.11	0.82	
**Plaque burden**				
None Non-calcified Mixt Calcified	32 (69.6) 3 (6.5) 7 (15.2) 4 (8.7)	25 (54.3) 6 (13.0) 7 (15.2) 8 (17.4))	0.19	
Total number of angles measured	114	121		
Number of angles per patient, mean +/- SD	2.46 +/- 0.55	2.58 +/- 0.62	0.25	
Sum of angles per patient, degrees, mean +/- SD	396 +/- 57	384 +/- 59	0.27	
Severe angulation	0	0		
Moderate angulation	1 (0.9)	2 (1.7)		
Mild angulation	113 (99.1)	119 (98.3)	1	

Results are expressed as n (%), unless specified otherwise. CCA: common carotid artery, SD: standard deviation

#### Procedural characteristics and post-procedural clinical outcomes

A left approach was chosen in three (6.5%) patients. All procedures were performed using the balloon-expandable transcatheter heart valves (THVs) of the Edwards SAPIEN family (SAPIEN 3 and SAPIEN 3 Ultra). Peri and post-procedural outcomes are shown in Table 3. No statistical comparison between the left and right sides was performed, given the small number of patients who underwent TAVI using the left CCA.

**Table 3:** Peri-procedural and post-procedural clinical outcomes according to the side of CCA that was used

	Left CCA (*n* = 3)	Right CCA (*n* = 43)
Peri-procedural outcomes		
Balloon-expandable THV Valve-in-valve TAVI Conversion to sternotomy	3 (100.0) 0 (0) 0 (0)	43 (100.0) 2 (4.6) 1 (2.3)
Myocardial infarction	0 (0)	1 (2.3)
**30-day post-procedural outcomes**		
All-cause mortality	0 (0)	0 (0)
PPM implantation	0 (0)	3 (7.0)
Stroke or TIA	0 (0)	1 (2.3)
Cardiac tamponade Major vascular complication Life-threatening bleeding Acute kidney injury	0 (0) 0 (0) 0 (0) 0 (0)	1 (2.3) 1 (2.3) 2 (4.6) 2 (4.6)

Results are expressed as n (%). CCA: common carotid artery, THV: transcatheter heart valve, TAVI: transcatheter aortic valve implantation, PPM: permanent pacemaker, TIA: transient ischemic attack

## Discussion

To our knowledge, our study is the first to compare the level of vessel tortuosity and plaque burden between the left CCA and the aortic annulus, as well as the right CCA and the aortic annulus, in patients undergoing TC-TAVI. Our exploratory study aimed to compare the two vascular pathways using validated and objective parameters.

The results of our study can be summarized as follows: (1) there was no significant difference regarding TIs and the number of angles between the left and right sides, (2) there was no significant difference in plaque burden between the two sides.

Although the use of auto-expandable and balloon-expandable THVs has been reported for TC-TAVI [8], the balloon-expandable Edwards SAPIEN family THVs (SAPIEN Ultra or SAPIEN 3) were exclusively used for TC-TAVI as well as all other non-TF TAVI interventions in our institution. Also, we had a preference for the right side because, in our experience, it provides an easier manipulation of the THV and its delivery system due to shorter distances between the access site and the aortic annulus, and a better alignment with the aortic root. This differs from the usual preference for the left-side approach in many other centers. However, we considered that if the right side exhibited a high TI, severe angulation, a high number of angles or a high plaque burden, accessing the left CCA should be favored.

The tortuosity of the supra-aortic vessels in patients undergoing TC-TAVI may be attributed to several factors. Firstly, advanced age and medical conditions, such as diabetes or hypertension, have been recognized as risk factors for arterial vessel tortuosity [11, 12]. Secondly, the attachment of carotid arteries to the skull and aorta can contribute to increased tortuosity due to age-related height reduction [11]. Finally, from a pathophysiological perspective, weakness in vessel walls related to abnormal elastin deposition or degradation has been incriminated in the development of arterial tortuosity [11, 13]. Considering that patients undergoing TAVI are typically elderly and often have multiple comorbidities, they are at a higher risk of presenting with extensive arterial tortuosity. This phenomenon has been well studied in TF-TAVI, where an increased iliofemoral tortuosity is known to be associated with local bleeding and complications when using the TF vascular access [17]. We extended this rationale to TC-TAVI, further analyzing if there was any difference between the left and the right CCAs, considering the ongoing debate regarding the preferred side for vascular access [9, 18].

TI and angulation of the CCAs have previously been assessed in different contexts, although not specifically in patients undergoing TC-TAVI. Using CTA imaging, Kamenskiy and colleagues compared CA geometry between healthy individuals (*n* = 15) and patients with atherosclerotic CA disease (*n* = 17) [14]. They observed a significantly higher tortuosity in the right CCA and internal CA (respectively *p* = 0.03 and *p* = 0.04). Although these findings do not align with our results, it is important to note that the study populations and methodologies differed significantly, and that the sample sizes were small in both studies, precluding any definitive conclusion. Additionally, Kamenskiy and colleagues did not take into account the aortic arch [14].

In contrast to carotid endovascular mechanical thrombectomy, where cervical vessel tortuosity has been shown to be associated with an increased risk of intervention failure or delay [19], we may speculate that high TI or severe angulation in TC-TAVI procedures could be risk factors for procedure failure or related complications. In our study, the incidence of postoperative complications was low, with one case of periprocedural stroke and two cases of life-threatening bleeding, all of which occurred during interventions using the right CCA, with no fatality.

It is unknown whether the use of a specific side for TC-TAVI could be associated with an increased risk of neurovascular complications. This question is particularly relevant given that strokes or transient ischemic attacks remain dreaded complications in TC-TAVI, because of direct CCA manipulation. Using data from 52 patients who underwent TC-TAVI, Faroux and colleagues found a higher number of silent cerebral ischemic lesions in the cerebral hemisphere ipsilateral to the CCA that was punctured (compared with the contralateral hemisphere), as assessed by systematic brain magnetic resonance imaging (MRI) exams [20]. Additionally, the use of large sheath/delivery systems was identified as an independent risk factor for ipsilateral ischemic cerebral embolism. However, due to the limited number of procedures using the right side (*n* = 2), a comparison between the right and left CCAs was not feasible. Furthermore, the study by Faroux and colleagues was not designed to analyze the relationship of the neurovascular complications with CA angulations. A larger study is needed to assess the peri-procedural clinical risk associated with the choice of the procedure side.

One significant factor that may influence the choice of vascular access side in TC-TAVI is arterial plaque burden. Previous studies have shown that plaque burden can vary based on cardiovascular risk factors and ethnic origin [21]. Our data did not show any significant difference in the number or type of arterial plaques between the right and left sides. While these factors could potentially influence the selection of the CCA side, our study did not find a specific pattern of plaque distribution or burden associated with either side. Another important factor is the extension of plaque: if the latter is very focal, vessel tortuosity is less a matter of concern since rigid guides may be able to strengthen the artery.

Finally, a last parameter that is difficult to quantify but likely of utmost importance in the surgeon’s decision-making process is their training or expertise.

Our study has several limitations that should be acknowledged. Firstly, it is retrospective and conducted at a single center, which limits the generalizability of our findings. Secondly, the relatively small sample size, although appropriate for an exploratory study, may have contributed to the lack of significant differences observed in our outcomes and prevents us from drawing definitive conclusions. Furthermore, the low number of procedures performed using the left side hinders the possibility of making a clinical comparison between left and right-side TC-TAVI interventions. Finally, the authors acknowledge that the outcomes which were analyzed, although objective, are not necessarily correlated with procedural complexity and clinical outcomes.

In conclusion, our study did not provide compelling objective evidence to support a preference for one specific access side over the other in TC-TAVI procedures. The integration of multiple factors, including arterial tortuosity and plaque burden, should guide the choice of the access side on an individual basis, taking into consideration a multidisciplinary approach. To further investigate the potential association between access side and procedural clinical outcomes, a larger study would be warranted.

## Data Availability

Data generated and analyzed in the present study are available from the corresponding author upon reasonable request.

## Abbreviations

CCA: Common carotid artery
CTA: Computed tomography angiography
TAVI: Transcatheter aortic valve implantation
TC: Transcarotid
TF: Transfemoral
TI: Tortuosity index
